# Diesel Exhaust Particles Induce Cysteine Oxidation and S-Glutathionylation in House Dust Mite Induced Murine Asthma

**DOI:** 10.1371/journal.pone.0060632

**Published:** 2013-03-29

**Authors:** Gerald B. Lee, Eric B. Brandt, Chang Xiao, Aaron M. Gibson, Timothy D. Le Cras, Lou Ann S. Brown, Anne M. Fitzpatrick, Gurjit K. Khurana Hershey

**Affiliations:** 1 Division of Asthma Research, Cincinnati Children’s Hospital Medical Center, Cincinnati, Ohio, United States of America; 2 Division of Allergy and Immunology, Cincinnati Children’s Hospital Medical Center, Cincinnati, Ohio, United States of America; 3 Division of Pulmonary Biology, Cincinnati Children’s Hospital Medical Center, Cincinnati, Ohio, United States of America; 4 Department of Pediatrics, Emory University School of Medicine, Atlanta, Georgia, United States of America; Virginia Commonwealth University, United States Of Ameirca

## Abstract

**Background:**

Diesel exhaust particle (DEP) exposure enhances allergic inflammation and has been linked to the incidence of asthma. Oxidative stress on the thiol molecules cysteine (Cys) and glutathione (GSH) can promote inflammatory host responses. The effect of DEP on the thiol oxidation/reduction (redox) state in the asthmatic lung is unknown.

**Objective:**

To determine if DEP exposure alters the Cys or GSH redox state in the asthmatic airway.

**Methods:**

Bronchoalveolar lavage fluid was obtained from a house dust mite (HDM) induced murine asthma model exposed to DEP. GSH, glutathione disulfide (GSSG), Cys, cystine (CySS), and s-glutathionylated cysteine (CySSG) were determined by high pressure liquid chromatography.

**Results:**

DEP co-administered with HDM, but not DEP or HDM alone, decreased total Cys, increased CySS, and increased CySSG without significantly altering GSH or GSSG.

**Conclusions:**

DEP exposure promotes oxidation and S-glutathionylation of cysteine amino acids in the asthmatic airway, suggesting a novel mechanism by which DEP may enhance allergic inflammatory responses.

## Introduction

Asthma is an inflammatory respiratory disorder with the greatest prevalence in developed societies [1]. One of the proposed explanations for this phenomenon is air pollution secondary to urbanization. Sharp increases in air pollution as a consequence of urbanization have been associated with similar increases in asthma prevalence [2]. Traffic-related air pollution is a major contributor to urban air pollution. Indeed, multiple epidemiological studies have linked the incidence and prevalence of asthma to exposure to traffic-related air pollution [3], [4], [5], [6]. Diesel exhaust particles (DEP) is a major component of traffic related air pollution and has been demonstrated to increase airway resistance and increase hyper-responsiveness in human subjects even with acute exposure [7]. Thusly, a wealth of data suggests that DEP exposure likely represents a significant contributor to the rising incidence and prevalence of asthma. However, the role of DEP in the pathogenesis of allergic asthma is not well understood.

The relationship between asthma and DEP may be secondary to DEP’s pro-inflammatory effects. By itself, DEP exposure induces neutrophilic influx into the lungs [7]. DEP exposure also enhances allergic sensitization and inflammation when co-administered with allergen in human nasal challenge studies [7]. This adjuvant activity of DEP on allergic inflammation has been associated with its ability to generate oxidative stress. DEP preparations containing more pro-oxidant organic compounds such as polyaromatic hydrocarbons induce greater sensitization to allergens and airway inflammation in murine asthma models [8]. Our lab has previously shown that a risk factor for persistent wheezing in DEP exposed infants is mutations in glutathione-S-transferase, an enzyme that catalyzes detoxification of reactive oxygen species by the thiol antioxidant glutathione (GSH) [9]. Furthermore, administration of the thiol antioxidant N-acetylcysteine prior to allergen exposure in mice has been shown to decrease the adjuvant activity of DEP on sensitization to ovalbumin [10]. Thus, thiol containing molecules appear to modulate the adjuvant effects of DEP.

However, to date few studies have examined the effect of DEP exposure on the thiol redox state of GSH and its precursor Cys in the asthmatic airway. GSH and Cys form a steady state with their respective oxidized disulfide forms, GSSG and CySS, and the GSH/GSSG and Cys/CySS redox couples are not in equilibrium [11]. In addition, oxidant conditions promote the formation of s-glutathionylated cysteine (CySSG) [12]. In order to assess the role of DEP exposure in oxidative stress asthma, we examined whether DEP exposure in combination with allergen alters Cys and/or GSH redox homeostasis in a house dust mite (HDM) induced murine asthma model.

## Methods

### Ethics Statement

This study was approved by the CCHMC Institutional Animal Care and Use Committee, and was conducted according to relevant institutional, national and international guidelines. The guide for The Care and Use of Laboratory Animals was used.

### Animals

**Figure 1:**
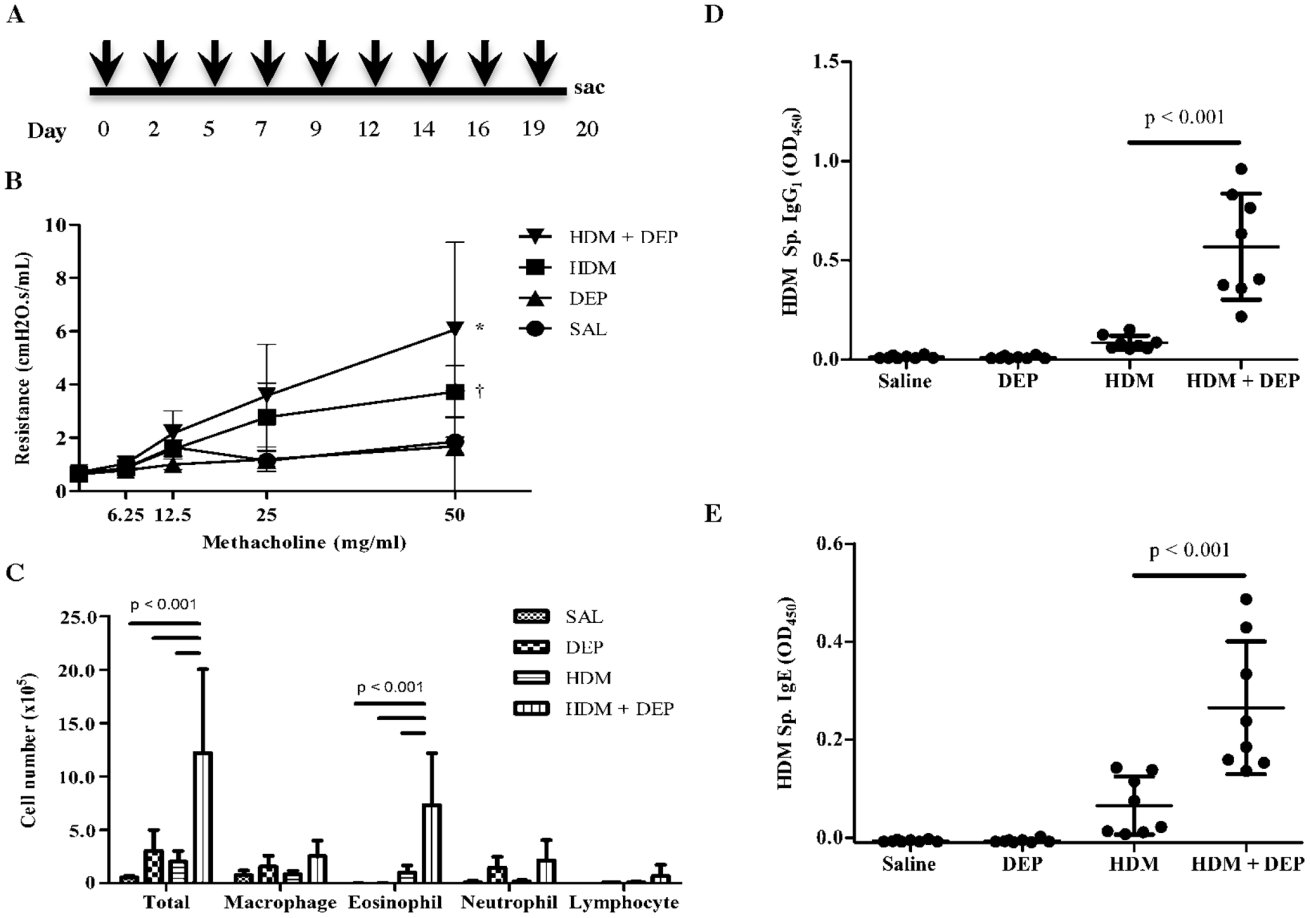
DEP co-exposure enhances HDM induced airway hyperresponsiveness (AHR), eosinophilic lung inflammation, and HDM sensitization. (A) Study protocol. 10 week old Balb/c male mice received nine intratracheal (IT) doses (arrows) over three weeks and were characterized 24 hours after the last dose. (B) AHR. (C), BALF cell counts. (D) Plasma HDM-specific IgG_1_ levels and (E) HDM-specific IgE levels . * p < 0.001 vs HDM, DEP and SAL, † p < 0.01 vs. DEP, SAL; Vertical bars represent the mean and SD for A,B and the horizontal bars represent the mean and SD for C,D. n =  8 mice per group.

All animal protocols were approved by the Institutional Animal Care and Use Committee. House dust mite extract (*Dermatophagoides pteronyssinus* [HDM]) was purchased from Greer Laboratories (Lenoir, NC). Diesel exhaust particles (DEP) were generated at the United States Environmental Protection Agency in the laboratory of M. Ian Gilmour using a 30 kW 4-cylinder Deutz BF4M1008 diesel engine connected to a 22.3 kW Saylor-Beall air compressor operating at a constant −20% load as previously described [8].10 week old Balb/c male mice, (purchased from Harlan Labs, Indianapolis, IN), were treated intratracheally with saline (SAL), 10 µg HDM, 150 µg DEP, or both HDM and DEP nine times over three weeks (Figure 1A). Twenty-four hours after the last treatment, airway hyperresponsiveness (AHR) was assessed using a flexiVent ventilator (Scireq, Montreal, QC, Canada) and ultrasonic nebulization of PBS (baseline) and increasing doses of methacholine (6.25, 12.5, 25, and 50 mg/ml; acetyl-β-methylcholine chloride, Sigma, St. Louis, MO).

### Bronchoalveolar lavage fluid collection and analysis

After the measurement of AHR, bronchoalveolar lavage was performed with 1 ml of Hank’s Buffered Salt Solution. Bronchoalveolar lavage fluid (BALF) was centrifuged at 200 x g for 7 minutes at 4°C. The supernatant was immediately removed and divided into 250 µl aliquots. To prevent auto-oxidation of the sample, one aliquot was preserved immediately in a 5% perchloric acid solution containing iodoacetic acid (6.7 µmol/L) and boric acid (0.1 mol/L) with 5 µmol/L γ-glutamyl-glutamate internal standard. Aliquots were stored at −80°C before analysis.

The cell pellet was resuspended in 250 ul of HBSS and the total cell numbers counted with a hemocytometer. Cells were spun onto slides and stained with the HEMA3 stain set (Fisher Scientific, Kalamazoo, Mich). Two hundred inflammatory cells were identified and counted the total number of each inflammatory cell type was calculated.

GSH, GSSG, Cys, CySS, and CySSG concentrations were measured in BALF supernatant by reverse-phase high-performance liquid chromatography after derivatization of the samples with dansyl chloride [13]. Derivatives were separated on a 10 mm Ultrasil amino-column with detection at 365 nm (WatersAlliance 2690, Waters Corporation, Milford, Mass). Fluorescence detection was recorded by 2 detectors (Waters 474, Waters Corporation, and Gilson 121, Gilson Inc, Middletown, Wis). GSH, GSSG, Cys, CySS, and CySSG were quantified relative to γ-glutamyl-glutamate by integration as previously described [13].

BALF cytokines levels were assessed by Luminex xMAP technology (Millipore, Billerica, MA), using Cytokine/Chemokine Panel I and following the manufacturer’s instructions. To assess total BALF IL-13 levels (free and IL-13R??2–bound), the IL-13 standard and samples were preincubated with 10 ng/ml murine IL-13R??2–Fc (R&D Systems) at 37°C for 1 hour. Anti-mouse IL-13 polyclonal antibody (1 mg/ml) was used for capture and biotinylated anti-mouse IL-13R??2 polyclonal antibody (0.5 mg/ml) for detection (R&D Systems).

### IgG_1_ and IgE ELISAs

Plasma was diluted 1∶5 for IgE and 1∶10,000 for IgG_1_. ELISAs were performed by using kits from BD Biosciences. For measurement of HDM-specific IgE and IgG_1_ levels, the wells in the assay plate were coated with 0.01% HDM (Greer Laboratories) overnight, and the remainder of the protocol was carried out according to the manufacturer’s instructions.

### HBEC culture and treatment

Normal human bronchial epithelial cells (HBEC) were kindly provided by Dr. Jeffrey Whitsett (Cincinnati Children’s Hospital Medical Center) [14]. HBEC were cultured in 0.1% gelatin pre-coated 6-well plates in Keratinocyte SFM media (Gibco) containing 50 µg/ml bovine pituitary extracts (BPE) and 5 ng/ml epidermal growth factor (EGF) at 37°C in 5% CO2. When 80% confluency was reached, cells were starved in the BPE/EGF-free media overnight, then treated with sonicated DEP at the dose of 5 µg/cm^2^ or HDM at the dose of 25 µg/ml and 100 µg/ml for 4 or 24 hours. RNA was extracted from cells using Trizol® (Invitrogen), treated with DNase and purified using RNeasy MinElute Kit (QIAGEN). Reverse transcription was done using Oligo-dT First-Strand cDNA Synthesis Kit (GE Healthcare) or First Strand cDNA Synthesis Kit (Invitrogen) using random hexamers. Quantitative PCR (qPCR) was done using SYBR Green Master Kit and LightCycler® 480 instrument (Roche Diagnostics) and primer sequences as follows: GAPDH: Forward 5′-GGGGAAGGTGAAGGTCGGAGTCA-3′ and Reverse 5′-AGCCTTGACGGTGCCATGGAAT-3′; CYA1PA: Forward 5′-ACCTCAGCCACCTCCAAGATCCC-3′ and Reverse 5′-GACAGTGCCAGGTGCGGGTT-3′; HMOX-1: Forward 5′-GCCAGCAACAAAGTGCAAG-3′ and Reverse 5′-GGCATAAAGCCCTACAGCAA-3′; NFE2L2: Forward 5′- CCAGCAGTGTCAGCTCAGGCTC-3′ and Reverse 5′-TGGGTGGTGGAGGTCCAAGGT-3′.

### Statistical Analysis

All statistical analysis was performed with PRISM software (GraphPad Software Inc, La Jolla, Calif). Statistical significance for AHR was assessed by using a 2-way ANOVA followed by a Bonferroni post-test. Otherwise, statistical significance was assessed by using 1-way ANOVA followed by a Tukey-Kramer post-test unless otherwise stated.

## Results

### DEP co-exposure enhances AHR, airway inflammation, and allergen sensitization induced by HDM

We established and characterized a mouse model of DEP exposure in HDM-induced asthma. DEP co-exposure with HDM increased AHR compared to HDM alone (p < 0.001; Fig 1B). DEP alone did not induce AHR. DEP co-exposure with HDM increased total inflammatory cell numbers and eosinophil numbers in the BALF when compared to HDM alone (Fig 1C). DEP co-exposure with HDM increased levels of HDM specific IgG_1_ and IgE in the plasma compared to HDM alone (Fig 1D,E).

**Figure 2:**
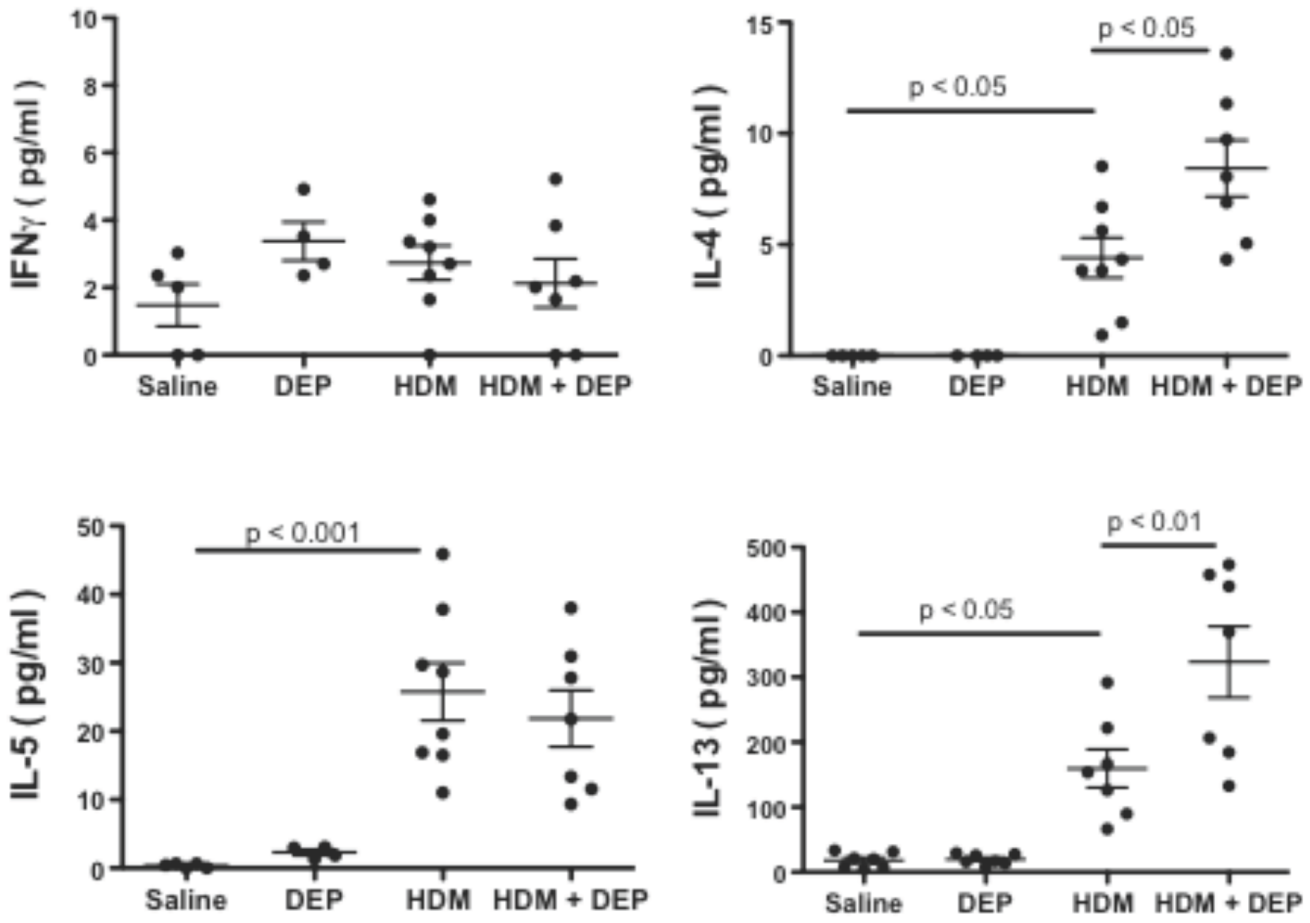
Effect of repeated exposure to DEP and/or HDM on cytokine levels in the BALF of mice subjected to the asthma model outlined in **Figure 1A**. IFNγ, IL-4, IL-5 and IL-13 were quantified by ELISA.

We determined cytokine levels in the BALF of treated mice (Figure 2). When DEP was given together with HDM, it resulted in significantly enhanced IL-4 and IL-13, but not IL-5 or IFNγ compared to HDM alone. DEP alone did not significantly induce the level of any Th1/Th2 cytokine measured in the BALF.

Taken together, this data demonstrates that DEP exposure has an adjuvant effect on HDM induced allergic responses including antibody production, lung inflammation, Th2 cytokines, and AHR.

### DEP co-administered with HDM does not alter the airway GSH redox state

**Figure 3:**
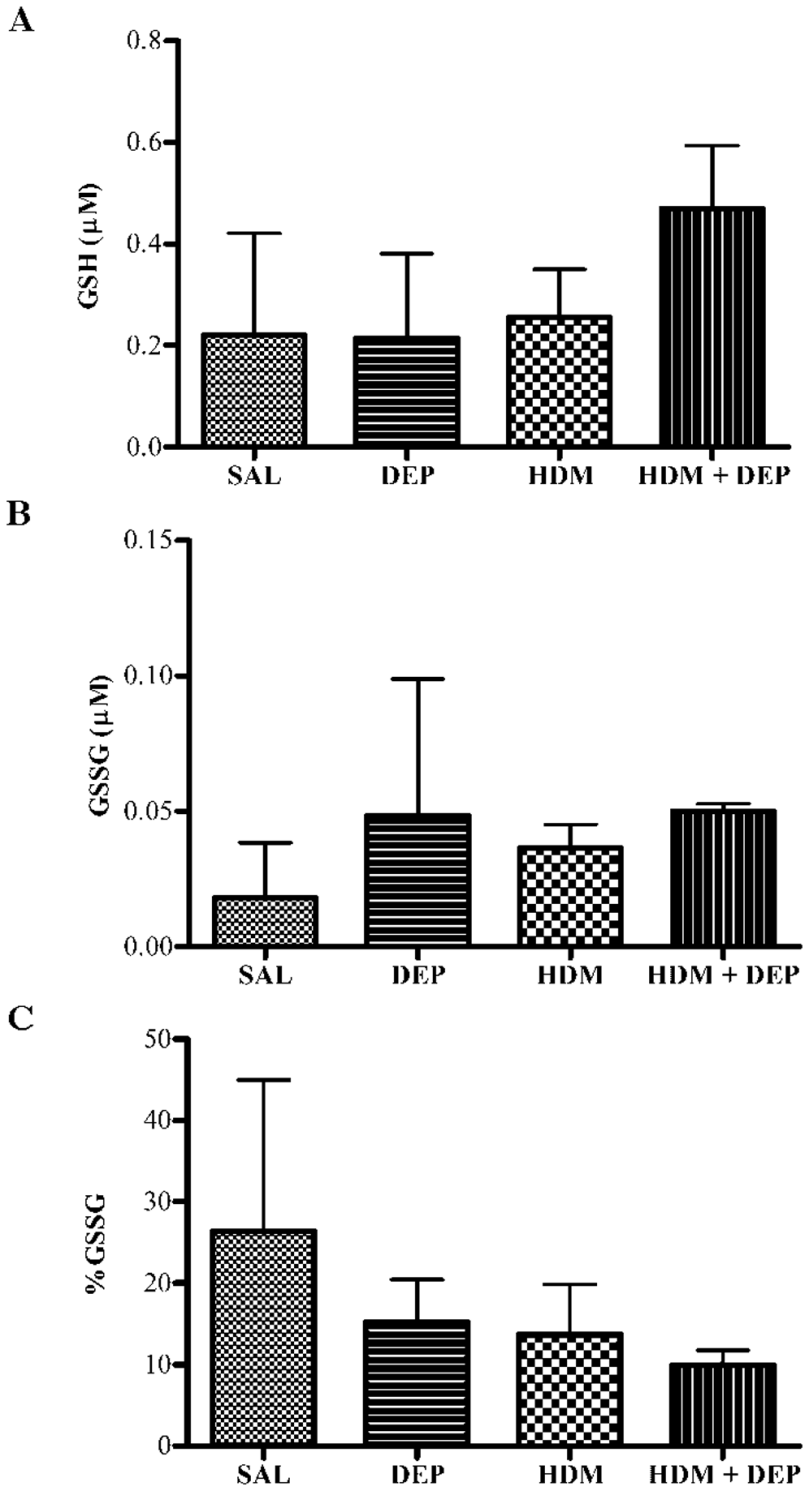
DEP co-administered with HDM does not significantly alter the airway GSH redox state. (A) GSH. (B) GSSG. (C)%GSSG. The horizontal bars represent the mean and SD for each group (n = 4−5 mice). Methacholine challenged mice were excluded.

GSH is an important thiol antioxidant in the lung which detoxifies reactive oxygen species by donating an electron and forming glutathione disulfide (GSSG) [15]. Therefore, an indicator of oxidative stress is an increase in the percentage of % GSSG, defined as GSSG/(GSH+GSSG) X100. DEP exposure did not alter GSH, GSSG, or % GSSG in the airway of mice with HDM-induced asthma (Figure 3A−C).

### DEP co-administered with HDM alters the airway Cys redox state

**Figure 4:**
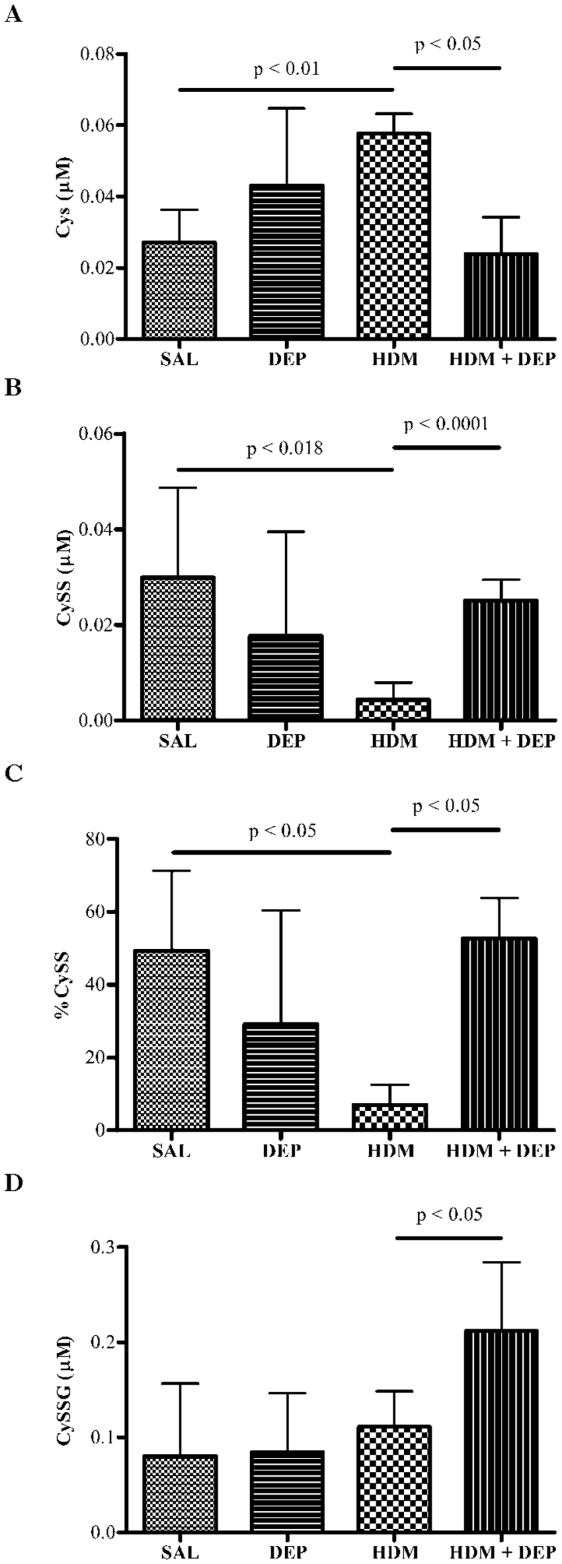
DEP co-administered with HDM induces cysteine oxidation in an asthma model. HDM + DEP exposed mice have decreased airway Cys (A), increased CySS (B), increased %CySS (C), and increased CySSG (D) when compared to HDM exposed mice. The horizontal bars represent the mean and SD for each group (n = 4−5 mice). Methacholine challenged mice were excluded. Panel B significant by t-test.

Cys is a precursor to GSH synthesis, which under oxidative conditions forms the disulfide cystine (CySS) or s-glutathionylated cysteine (CySSG). The ratio of Cys to CySS is independent of the GSH/GSSG couple and therefore represents an independent indicator of oxidative stress [11]. HDM exposure alone significantly increased airway Cys and decreased CySS. In contrast, DEP co-exposure with HDM decreased total airway Cys and increased both oxidized CySS and CySSG (Figure 4).

### Exposure of Human Bronchial Epithelial Cells to DEP but not HDM induces oxidative response genes

**Figure 5:**
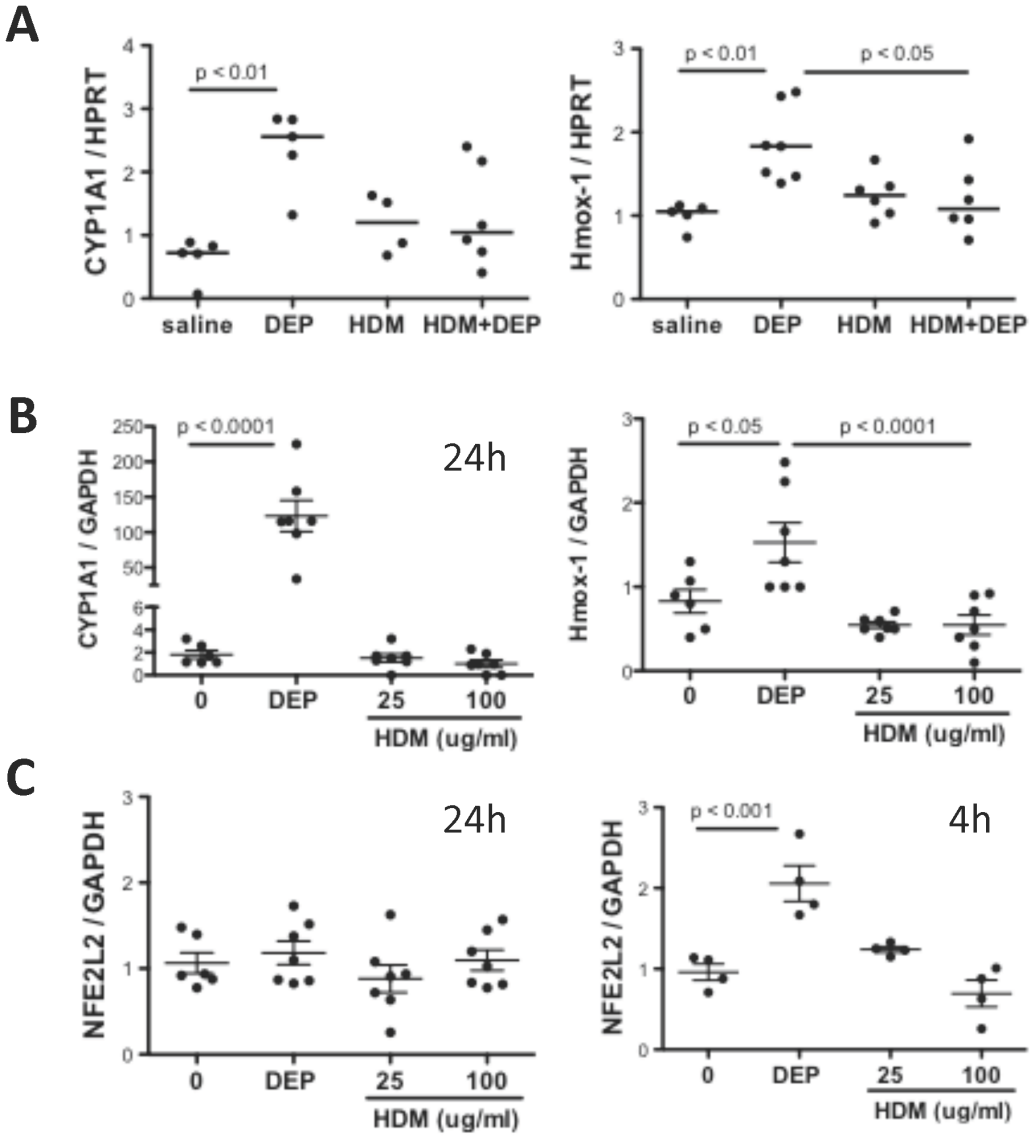
Effect of DEP and HDM exposure on induction of oxidative stress response genes. (A) Expression levels of the oxidative stress genes CYP1A1 and HMOX1 in murine lungs 24h after the 9^th^ IT challenge with saline, DEP, HDM or HDM+DEP as indicated. mRNA levels were determined by quantitative PCR and normalized to HPRT levels. Human bronchial epithelial cells were treated with media, DEP (5 µg/cm^2^) or HDM (25 or 100 µg/ml) for 4h or 24h and then (B) mRNA levels of CYP1A1 and HMOX1 were assessed in HBEC at 24h. (C) mRNA levels of NFE2L2 were assessed in RNA derived from HBEC at 4h and 24h following exposure. mRNA levels were determined by quantitative PCR and normalized to GAPDH levels.

Our data thus far demonstrated that DEP induce cysteine oxidation and S-glutathionylation when co-exposed with HDM, however, neither DEP nor HDM alone had these effects. In order to further define the effect of DEP and HDM on oxidative stress, we determined the effect of DEP and HDM exposure on the induction of genes important in the response to oxidative stress, NFE2L2, HMOX1, and CYP1A1. In the animal model (Figure 1A), only exposure to DEP alone induced CYP1A1 and HMOX1 (Figure 5A). HDM alone or in combination with DEP failed to induce CYP1A1 and HMOX1 (Figure 5A). We confirmed these results *in vitro*, using human bronchial epithelial cells (HBEC) stimulated with DEP or HDM. As shown in Figure 5B, CYP1A1 and HMOX1 genes were induced by DEP, but not by HDM even at a high dose of 100 µg/ml. NFE2L2 levels were significantly increased in HBEC after 4h of DEP exposure but not at 24 hours (Figure 5C), indicating that DEP induction of NFE2L2 is an early transient event.

## Discussion

**Figure 6:**
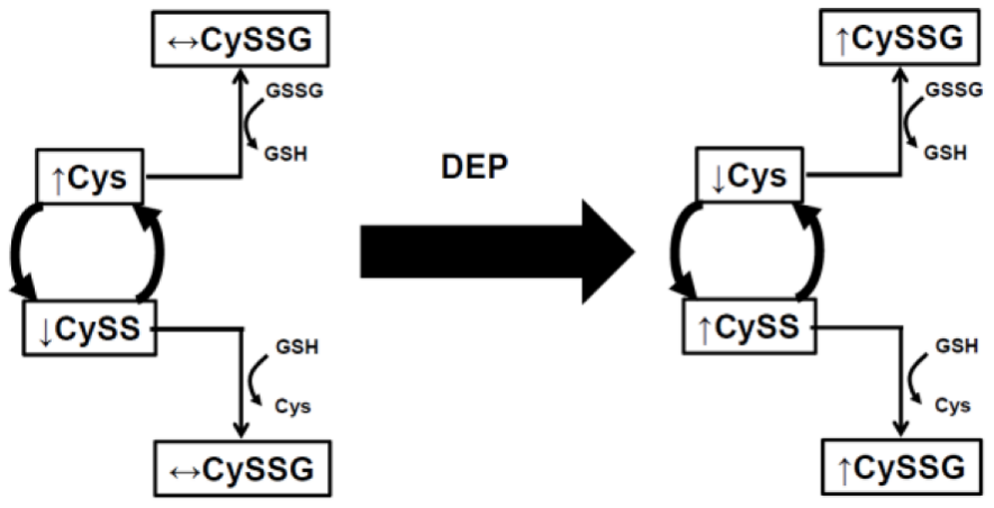
Schematic showing proposed effects of DEP exposure on the BALF GSH and Cys redox states in HDM-induced murine asthma.

There is increasing evidence that DEP exposure is a significant contributor to prevalence and severity of asthma. However, the role of DEP in the pathogenesis of allergic asthma is not well understood. This study demonstrates that DEP synergizes with HDM to specifically alter the cysteine redox state towards a more oxidized state and increases s-glutathionylated cysteine in HDM induced asthma (Figure 6). In contrast, glutathione redox state was not altered by DEP exposure in this model. Since the GSH and Cys redox couples are not in equilibrium, the findings suggest that the Cys redox state is a biomarker of DEP induced oxidative stress.

The observed alterations in the Cys redox state suggest that DEP could also induce redox modification of cysteine residues on proteins. Redox modification of proteins has been demonstrated to alter signal transduction, transcription factor binding affinity, and receptor activation/deactivation [12]. The formation of mixed disulfides on proteins also can modify the inflammatory response. Protein s-glutathionylation has been shown to modify NF-kappaB activation in lung epithelial cells [16]. Thus, DEP adjuvant effects may be partially due to pro-oxidant disruption of redox-sensitive pathways that regulate inflammation.

An interesting finding was the increased glutathione and cysteine concentration in the BALF after allergen exposure. Glutathione transport to the extracellular space has been shown to occur as a first defense against an oxidant stress [17]. The increased glutathione observed in the BALF is in contrast to the decreased glutathione and depressed GSH/GSSG ratio reported in the BALF of severe asthmatics [18]. There are a few explanations for this discrepancy. First, this mouse model is a relatively short-term asthma model. Since the physiological response to oxidative stress is hierarchal, it is possible that our model represents the early compensatory responses to oxidant stress without engaging the more advanced stages seen in severe asthma [17]. Also, the allergic status of the severe asthmatics was not reported in this study [18], which makes it difficult to compare these findings to our allergic asthma model. These severe asthmatics also were taking corticosteroid medications, which could alter the GSH and Cys redox state. Finally, GSH content in the plasma is much higher in rodents than in humans, while plasma Cys is more alike [19].

In summary, we found that DEP exposure induces oxidative stress that specifically oxidizes the cysteine oxidative state in the epithelial lung fluid in allergic asthma and increases S-gluathionylation of cysteine. Consistent with this, DEP exposure resulted in the induction of oxidative stress response genes in human bronchial cells and in mouse lungs supporting that DEP contributes to increased oxidative stress. This increased oxidative stress, along with the increase in Th2 cytokines observed following DEP and HDM co-exposure, may be responsible for the observed health impact of DEP on asthma prevalence and severity. Since s-glutathionylation of cysteine was only seen in the DEP+HDM treated mice, it is possible that s-glutathionylated cysteine could be a specific biomarker for of DEP pollution exposure in allergic asthma. This is highly relevant because 45% of the US population resides in zones of high DEP exposure [20]. Although previous trials using anti-oxidant therapy have not been successful in asthma [21], careful subject selection using s-glutathionylated cysteine as a selection criteria may better phenotype and select for patients who would specifically benefit from antioxidant therapy. In addition, thiol antioxidants such as N-acetylcysteine may be a more effective therapy for these patients.
